# Is Ready‐To‐Use AbobotulinumtoxinA More Effective Than OnabotulinumtoxinA for Glabellar Lines? A Randomized, Controlled, Triple‐Blinded Clinical Trial

**DOI:** 10.1111/jocd.70816

**Published:** 2026-03-26

**Authors:** Jacqueline Rosa Gonçalves, Ligia Figueiredo Valesan, Ana Claudia Laureano Navarro Shishido, Alfonso Sanchez‐Ayala, Ana Claudia Carbone, Mariana Barbosa Câmara‐Souza, Giancarlo De la Torre Canales

**Affiliations:** ^1^ Department of Dentistry Ingá University Center, Uningá Paraná Brazil; ^2^ Department of Dentistry Federal University of Santa Catarina Florianopolis Brazil; ^3^ Department of Dentistry University of Ponta Grossa Ponta Grossa Paraná Brazil; ^4^ Department of Dentistry Sao Leopoldo Mandic University Campinas Brazil; ^5^ Egas Moniz Center for Interdisciplinary Research (CiiEM); Egas Moniz School of Health & Science Caparica, Almada Portugal; ^6^ Division of Oral Diagnostics and Rehabilitation, Department of Dental Medicine Karolinska Huddinge Sweden

**Keywords:** botulinum toxin type, glabellar lines, onabotulinumtoxinA, ready‐to‐use abobotulinumtoxinA

## Abstract

**Background:**

Most commercially available botulinum toxin A (BoNT‐A) formulations are lyophilized or vacuum‐dried powders requiring reconstitution. Recently a ready‐to‐use formulation (RTUaboBoNT‐A) has been introduced. Although its efficacy in reducing glabellar lines has been reported, comparative data with powder onabotulinumtoxinA (OnaBoNT‐A) remain limited.

**Aims:**

To evaluate the efficacy, durability, and safety of RTUaboBoNT‐A compared with OnaBoNT‐A for moderate to severe glabellar wrinkles:

**Methods:**

Forty women (25–50 years) were randomly allocated to receive either OnaBoNT‐A (*n* = 20) or RTUaboBoNT‐A (*n* = 20) at equivalent doses in the procerus and corrugator muscles. Outcomes included Electromyography activity (EMG), Merz 5‐point glabellar lines scale, FACE‐Q satisfaction scores, perceived age and pain intensity (VAS). Assessments were performed at baseline and 1, 2‐, 3‐, 4‐ and 5‐months post‐injection. Statistical analysis included two‐way repeated‐measures ANOVA with Bonferroni's post hoc, chi‐squared tests and independent Student's t‐tests.

**Results:**

No significant intergroup differences were observed in EMG activity of the assessed muscles across follow‐ups (*p* > 0.05). Glabellar lines severity was comparable between groups at rest and maximum contraction throughout the study (*p* > 0.05). Higher satisfaction scores were observed at 2‐months in the OnaBoNT‐A group (*p* = 0.01). Pain during injection was greater with RTUaboBoNT‐A (*p* = 0.02). No intergroup differences were found in perceived age (*p* > 0.05).

**Conclusion:**

The RTUaboBoNT‐A demonstrated similar efficacy, durability, and safety profile to OnaBoNT‐A for the treatment of glabellar lines. With minor differences in satisfaction and injection disconfort.

## 1 Introduction

Facial expressions play a central role in social interaction, and changes in the glabellar region are particularly relevant to how individuals perceive their own appearance and emotional expression [1, 2, 3]. Prominent glabellar lines are frequently associated with negative self‐perception and reduced aesthetic satisfaction, making this area one of the most treated targets in facial rejuvenation [4]. Among available interventions, botulinum toxin type A (BoNT‐A) injections are considered the reference standard for managing dynamic glabellar wrinkles [5, 6, 7].

BoNT‐A is a neurotoxin produced by *Clostridium botulinum* , with serotype A being the most extensively investigated and clinically applied variant [8]. Its widespread adoption is reflected in global practice patterns, as highlighted by the 2024 International Survey on Aesthetic/Cosmetic Procedures, which identified BoNT‐A as the most frequently performed non‐surgical aesthetic treatment worldwide [9]. Independent of formulation, BoNT‐A products have consistently demonstrated robust safety and efficacy profiles for the treatment of glabellar lines [1, 6, 7].

Traditionally, commercially available BoNT‐A formulations are supplied as lyophilized or vacuum‐dried powders that require reconstitution with 0.9% sodium chloride prior to administration [10]. Although effective, this preparation process may introduce practical challenges in routine clinical practice, including variability in dilution techniques, dosing accuracy, preparation time, and injector‐dependent differences, all of which may influence aesthetic outcomes [11, 12]. These concerns have stimulated interest in alternative formulations capable of simplifying clinical workflows while maintaining therapeutic performance.

In response to these considerations, a ready‐to‐use liquid formulation of abobotulinumtoxinA (RTU‐aboBoNT‐A; Alluzience, Ipsen/Galderma, Lausanne, Switzerland) has been developed. This formulation consists of a stabilized aqueous solution containing histidine, sucrose, polysorbate‐80, sodium chloride, hydrochloric acid, and water for injection, without animal‐ or human‐derived excipients. RTU‐aboBoNT‐A can be stored at room temperature for up to 12 h prior to use without compromising its pharmacological properties [13]. Clinical studies have demonstrated its efficacy and safety for the treatment of moderate to severe glabellar lines, including sustained effects following repeated administrations [14, 15], as well as high levels of patient satisfaction [16]. Moreover, ease of use has been identified as a relevant advantage when compared with powder‐based BoNT‐A formulations [17].

Despite these favorable characteristics and clinical outcomes, RTU‐aboBoNT‐A remains a relatively recent addition to aesthetic practice, and evidence derived from randomized clinical trials is still limited [15, 16]. Comparative investigations with other BoNT‐A formulations are restricted and mainly consist of randomized trials comparing RTU‐aboBoNT‐A with powdered abobotulinumtoxinA or onabotulinumtoxinA reporting similar effectiveness and safety based on subjective outcome measures [14, 17]. Furthermore, in a randomized, triple‐blinded clinical trial conducted by our group, no significant differences were observed between RTU‐aboBoNT‐A and powdered abobotulinumtoxinA after 4 months of follow‐up with respect to muscle electrical activity, glabellar wrinkle severity, and patient satisfaction with the treatments. However, RTU‐aboBoNT‐A was associated with significantly higher pain intensity at the time of application [18].

Taken together, these findings highlight the need for further well‐designed comparative studies to better characterize the clinical performance, tolerability, and patient‐centred outcomes of RTU‐aboBoNT‐A relative to established BoNT‐A formulations. Therefore, the present randomized, triple‐blind clinical trial compared the effectiveness, durability, and safety of ready‐to‐use abobotulinumtoxinA (Alluzience) and onabotulinumtoxinA (Botox) for the treatment of moderate to severe glabellar lines in women.

## 2 Methods

This randomized, triple‐blind clinical trial was conducted in accordance with the Declaration of Helsinki and received approval from the Research Ethics Committee of Uningá University (CAAE: 83084224.4.0000.5220). Prior to enrolment, all participants were fully informed about the study objectives and procedures and provided written informed consent. The trial was carried out at a private specialized aesthetic clinic between March and December 2025. Reporting of this study followed the Consolidated Standards of Reporting Trials (CONSORT) guidelines.

### 2.1 Participants

The study population consisted exclusively of Brazilian women aged 25–50 years presenting with dynamic glabellar lines graded II to IV according to the Merz 5‐point scale. Participants were excluded if they had received prior BoNT‐A injections for any indication, undergone aesthetic procedures in the upper third of the face, or presented with autoimmune or neuromuscular disorders. Additional exclusion criteria included receipt of any vaccine within 3 months before study entry and current use of medications known to interfere with neuromuscular transmission.

Sample size estimation was performed using G*Power software (version 3.1.9.2, Kiel, Germany), based on pilot data derived from electromyographic recordings of the procerus muscle. Calculations were conducted for a repeated‐measures ANOVA with within–between interaction, assuming an alpha level of 0.05, statistical power of 80%, two treatment groups, six repeated measurements, and an effect size f of 0.41. The minimum required sample was estimated at 36 participants. To compensate for a potential dropout rate of approximately 10%, a total of 40 participants were recruited.

### 2.2 Study Protocol

Participants attended a total of six study visits. During the initial visit, eligibility was assessed according to the predefined inclusion and exclusion criteria, and detailed explanations regarding the study procedures were provided. Eligible participants were informed that a single BoNT‐A injection session would be performed using one of the two study products. At the second visit, baseline assessments were completed, randomization was finalized, and BoNT‐A injections were administered. Follow‐up evaluations were conducted at 1, 2, 3, 4, and 5 months after treatment. At each follow‐up visit, all outcome measures were systematically collected. Throughout the study, participants, outcome assessors, and the clinician administering the injections remained blinded to the allocated treatment.

### 2.3 Randomization and Blinding

Random allocation was generated using a computer‐based randomization system (http://www.randomization.com/) with block sizes of two, under the responsibility of a technician who had no further involvement in the trial. Allocation concealment was ensured by placing the assigned BoNT‐A product for each participant in an opaque, sealed envelope. The randomization list was inaccessible to investigators until completion of data collection. Consequently, treatment allocation and block size were unknown to the injector (L.F.V.), the outcome assessor (J.R.G), and the participants. Immediately before treatment administration, a third investigator, solely responsible for product preparation, opened the envelope and prepared the syringes. Participants were randomized in onabotulinumtoxinA group (OnaBoNT‐A): *n* = 20 and ready‐to‐use abobotulinumtoxinA group (RTU‐aboBoNT‐A): *n* = 20. Syringes were labeled with participant identification codes and placed in the treatment room prior to the entry of both the participant and the injecting clinician. Both BoNT‐A formulations were colorless and visually indistinguishable.

### 2.4 Interventions

Injection procedures followed the specific consensus recommendations for each product [19, 20]. For the onabotulinumtoxinA group (Botox, Allergan, Irvine, CA, USA), vials containing 100 U were reconstituted with sterile 2mls of 0.9% saline solution according to standard clinical practice. The ready‐to‐use abobotulinumtoxinA group received Alluzience (Galderma SA, Lausanne, Switzerland), supplied as a solution for injection at a concentration of 200 U/mL. Each vial contained 0.625 mL of deliverable volume of solution. For both groups, a total dose equivalent to 20 U was administered in the glabellar region, distributed across five standardized injection points (4 U per point). The procerus muscle was injected at a single midline point at the level of the medial canthal ligaments, with the needle inserted perpendicular to the skin until bone contact. The corrugator supercilii muscles were injected bilaterally at two points per muscle: one superficial injection directed inferomedially at approximately 45° at the tail region, and one deep injection at the muscle origin with a perpendicular approach. No supplementary injection sites were used. Preparation of the study products was performed by a trained investigator, while all injections were administered by an experienced clinician specialized in aesthetic injectable procedures. Injections were delivered using 1‐mL syringes fitted with 27.5‐gauge, 6‐mm needles.

### 2.5 Outcomes

Outcome assessments were conducted at baseline and at 1, 2‐, 3‐, 4‐, and 5‐months post‐treatment. The objective assessment electromyographic activity of the procerus and corrugator supercilii muscles constituted the primary outcome. Subjective secondary outcomes included evaluations of glabellar line severity (Merz 5‐point scale) [21], patient‐reported satisfaction and age perception (FACE‐Q “Appraisal of Lines Between Eyebrows and FACE‐Q patient‐perceived age Visual Analogue Scale”) [22], and subjective pain intensity during injection session (Visual Analogue Scale (VAS)) [23].

#### 2.5.1 Electromyography (EMG)

Surface electromyographic recordings were obtained using a four‐channel EMG system (Miotool NG USB, Porto Alegre, Brazil; bandwidth 10–700 Hz; sampling rate 3000 Hz; resolution 2.44 μV/bit) operated by a single calibrated examiner. Bipolar Ag/AgCl surface electrodes (3.2 × 2.8 cm) were positioned over the procerus muscle at the cranial end of the nasal bones and over the corrugator supercilii muscles at the midpoint of the superior eyebrow margin. A reference electrode was placed on the manubrium of the sternum [24]. Muscle activity was recorded during maximum voluntary contraction (MVC), with participants instructed to perform maximal glabellar frowning for 5 s. Three contractions were recorded for each muscle, with a 2‐minute rest interval between trials to minimize fatigue. Signals were sampled at 1000 Hz, band‐pass filtered between 20 and 500 Hz, and processed to obtain root mean square (RMS) values using MiotecSuite software (version 1.0). The mean RMS value from the three contractions was used for statistical analysis.

#### 2.5.2 Severity of Glabellar Lines

Glabellar line severity was assessed both at rest and during MVC using the Merz 5‐point scale, which classifies wrinkles from 0 (no lines) to 4 (very severe lines). Ratings were based on visual self‐assessment by the participants and by the assessor [21].

#### 2.5.3 Patient‐Reported Satisfaction With Treatment

Patient satisfaction was evaluated using the FACE‐Q scale, “FACE‐Q Appraisal of Lines Between Eyebrows [25] and FACE‐Q patient‐perceived age Visual Analogue Scale” [26]. For the first one, participants rated how bothered they felt by the glabellar region during the previous week on a 4‐point scale ranging from “not at all” to “extremely.” Raw scores were summed and converted to a standardized 0–100 scale, with higher scores indicating greater satisfaction. For the second one, participants reported the age they perceive relative to their actual age prior to and after treatment. This scale ranges from 15 years older to 15 years younger with increments of 1 year. Authorization to use the FACE‐Q instrument for non‐profit academic research was obtained by the investigators.

#### 2.5.4 Subjective Pain Intensity

Pain associated with the injection procedure was assessed immediately after treatment using a 10‐cm VAS, anchored by “no pain” and “worst imaginable pain.” Participants marked the point that best represented their perceived pain intensity [23].

#### 2.5.5 Adverse Events

Participants were asked to report any adverse events related to the injections in the first follow‐up assessment.

### 2.6 Statistical Analysis

Statistical analyses were performed using IBM SPSS Statistics (version 25; IBM Corp., Armonk, NY, USA). All tests were two‐tailed, with a significance level set at *α* = 0.05 and statistical power of 80%. Data distribution and variance homogeneity were examined using the Shapiro–Wilk and Levene tests, respectively. All analyses were based on an Intention To Treat (ITT) analysis. Time‐ and treatment‐related effects on electromyographic activity and FACE‐Q scores were analyzed using two‐way repeated‐measures ANOVA, with Bonferroni correction applied for post hoc comparisons. Independent Student's *t*‐tests were used to compare age and VAS scores between groups, while wrinkle severity distributions were analyzed using chi‐square tests.

## 3 Results

### 3.1 Study Population

**FIGURE 1:**
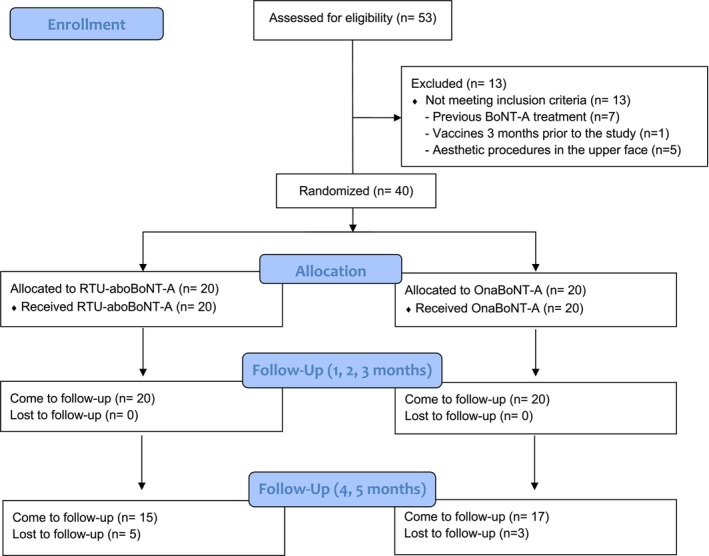
CONSORT Flaw diagram of the included patients.

A total of 53 women were assessed for eligibility and 40 were included in the analysis, with a mean age of 37.22 ± 7.08 years. Most of the patients completed the study, except for three patients in the OnaBoNT‐A and five patients in the RTU‐aboBoNT‐A group (Figure 1). Age distribution did not differ significantly between groups (*p* > 0.05). Baseline severity of glabellar lines in maximum contraction was comparable between groups, with no statistically significant differences observed (*p* > 0.05). In the OnaBoNT‐A group, wrinkle severity was classified as very severe in 65% of participants (13), severe in 20% (4), and moderate in 15% (3). Corresponding proportions in the RTU‐aboBoNT‐A group were 55% very severe (11), 30% severe (6), and 15% moderate (3).

### 3.2 Electromyographic Activity

**FIGURE 2:**
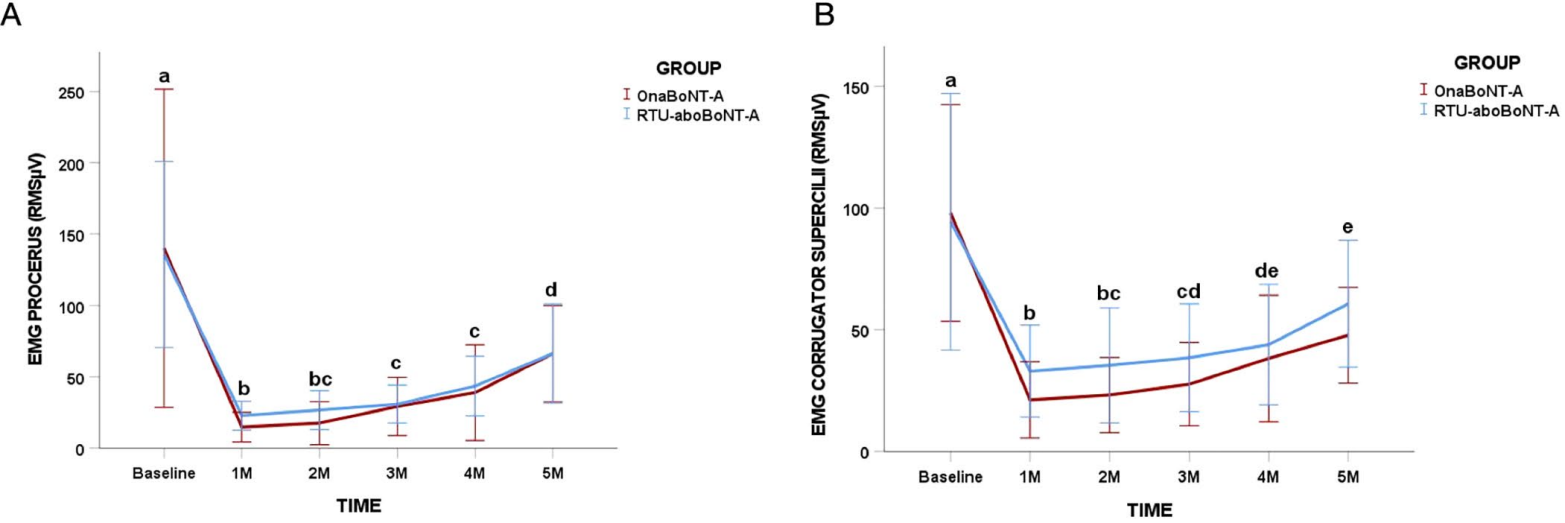
Changes in root mean square scores (RMS μV) in maximum voluntary contraction (MVC) of (A) procerus and (B) corrugator supercilli according to toxin group and timepoints. Different lower‐case letters mean significant intra‐group differences at 0.05. No inter‐group differences were found in the study.

Within‐group analyses demonstrated a significant decrease in electromyographic activity of both the procerus and corrugator supercilii muscles at all post‐treatment follow‐up points compared with baseline in both treatment groups (*p* = 0.001; Figure 2A,B). When electromyographic values obtained at the 3‐month evaluation were compared with those from subsequent follow‐ups, no further significant reductions were detected in either muscle for either group (*p* > 0.05). Between‐group comparisons revealed no statistically significant differences between treatments for electromyographic activity of the procerus or corrugator supercilii muscles at any assessment time point (*p* > 0.05; Figure 2A,B).

### 3.3 Glabellar Lines Severity

**FIGURE 3:**
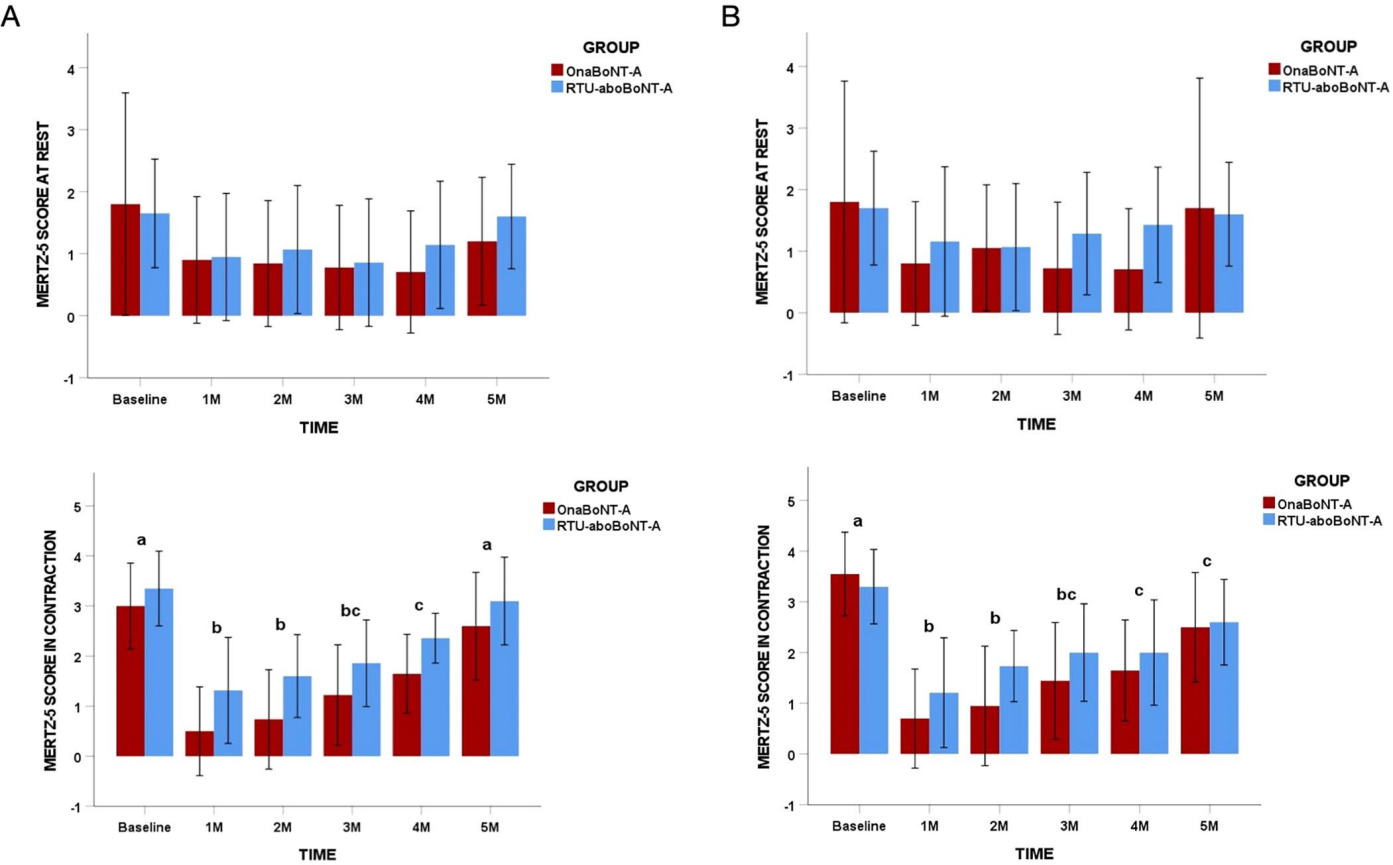
Frequency of Merz scores according to toxin group and timepoints. Column (A) assessor reports and column (B) patients report. Different lower‐case letters mean significant intra‐group differences at 0.05. No inter‐group differences were found in the study.

Intra‐group analyses indicated a significant reduction in glabellar lines severity during maximum contraction for both treatment groups at the 1‐, 2‐, 3‐, and 4‐month follow‐up assessments when compared with baseline, according to both patient self‐assessments and evaluator ratings (*p* = 0.0001; Figure 3). At the 5‐month evaluation, a statistically significant reduction relative to baseline remained evident only in patient‐reported assessments (*p* = 0.001). No statistically significant differences between treatment groups were observed for glabellar lines severity, either at rest or during contraction, at any time point throughout the study, based on both patient and evaluator assessments (*p* > 0.05; Figure 3A,B).

### 3.4 Patient Satisfaction With Treatment

**FIGURE 4:**
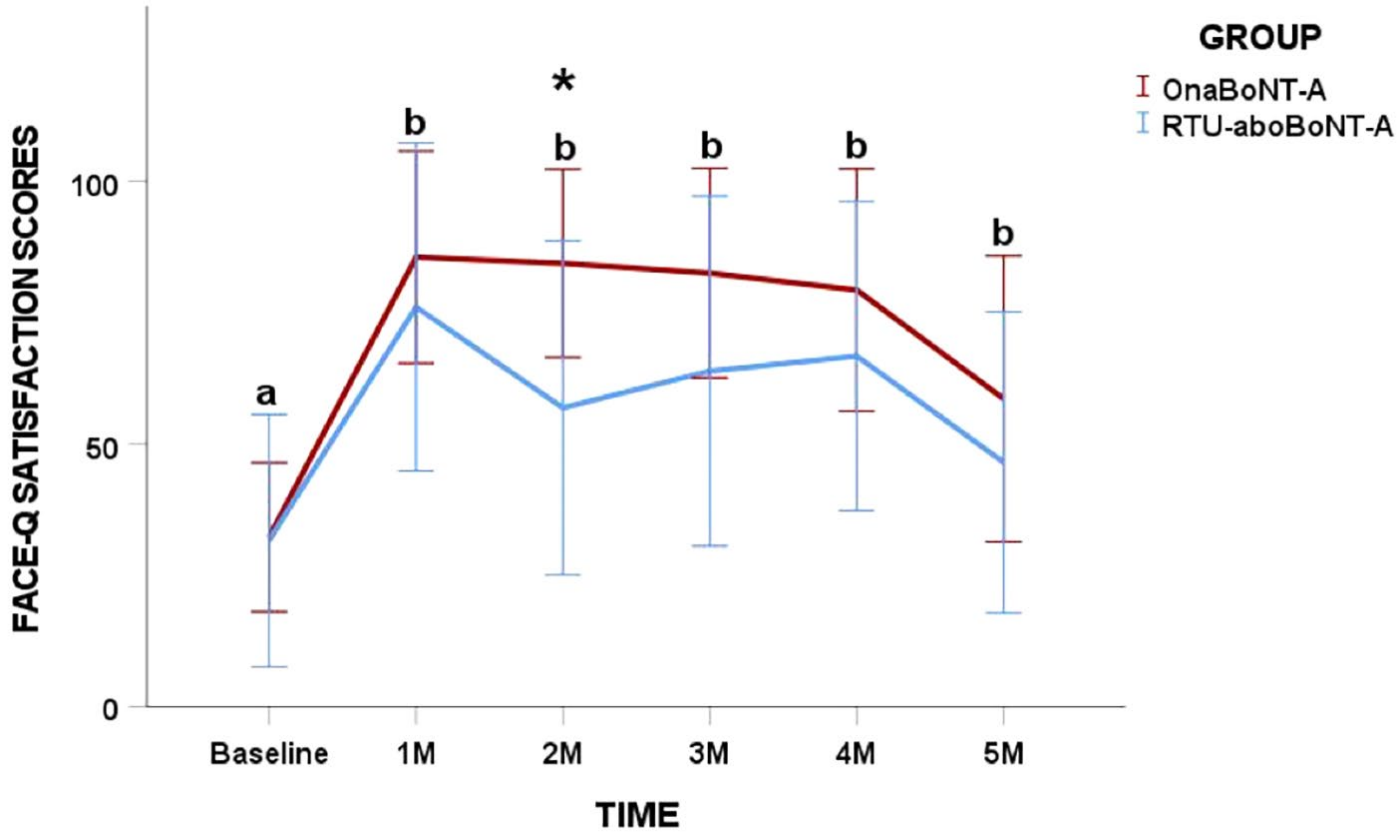
Post‐neuromodulation changes in Face‐Q Satisfaction scores for each toxin group in different timepoints. Different lower‐case letters mean significant intra‐groups differences at 0.05. *Inter‐group significant differences (*p* < 0.05).

Within‐group analyses demonstrated a significant increase in patient satisfaction scores for both treatment groups at all follow‐up assessments when compared with baseline (*p* = 0.001). In between‐group comparisons, significantly higher satisfaction scores were observed in the OnaBoNT‐A group exclusively at the 2‐month follow‐up relative to the RTU‐aboBoNT‐A group (*p* = 0.01; Figure 4).

**FIGURE 5:**
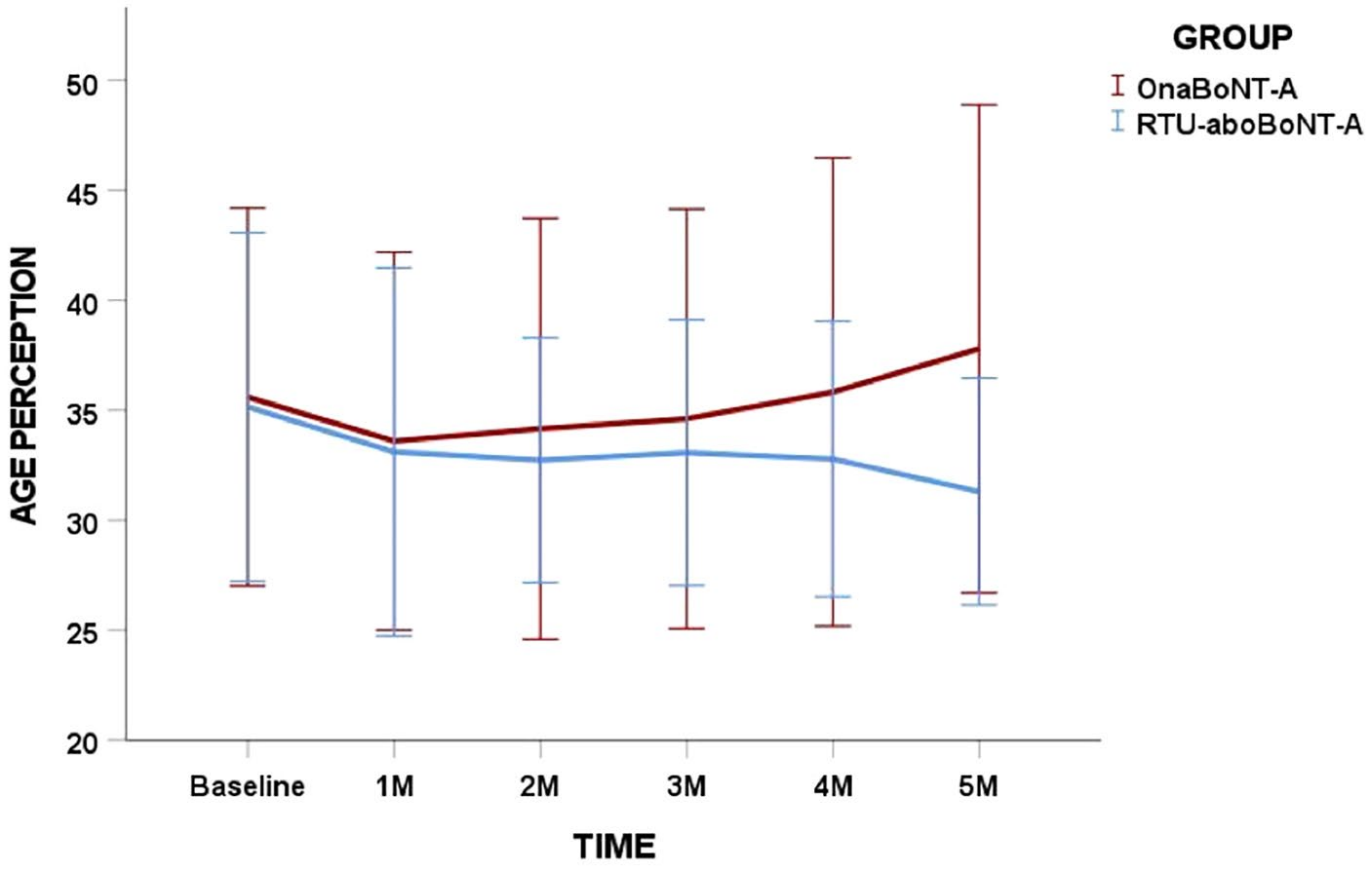
Post‐neuromodulation changes in FACE‐Q Perceived Age Visual Analogue Scale scores for each toxin group over time. No intra and inter‐group differences were found in the study.

No statistically significant differences were identified in perceived age, either within groups over time or between groups at any assessment point (*p* > 0.05; Figure 5).

### 3.5 Pain Intensity

VAS scores showed a statistically significant difference between groups, with participants treated with RTU‐aboBoNT‐A reporting higher pain scores (4.33 ± 2.07) compared with those receiving OnabotulinumtoxinA (2.25 ± 2.63; *p* = 0.02).

### 3.6 Adverse Events

No serious adverse events were reported for any of the participants in both groups throughout the study. However, three patients in the OnaBoNT‐A group reported itching at the injection site (1 week), cutaneous rash at the malar region (2 weeks), and under‐eye bags (1 week), respectively. For RTU‐aboBoNT‐A group one patient reported itching at the injection site. All adverse events resolved spontaneously without the need for additional intervention.

## 4 Discussion

The present study compared the clinical performance of RTU‐aboBoNT‐A and OnaBoNT‐A for the treatment of moderate and severe glabellar lines in a female population. Our study found that both formulations demonstrated comparable efficacy and safety profiles, with consistent diminution of muscle EMG activity, improvements in glabellar lines, and enhanced patient‐reports of satisfaction with the treatments. However, even though both formulations presented positive results, patients did not perceive themselves younger after the treatment but reported significantly more pain when the RTU‐aboBoNT‐A was injected. Overall, the findings indicate that RTU‐aboBoNT‐A achieves therapeutic results similar to those of the established lyophilized formulation.

Electromyographic analysis showed that both treatments produced significant reductions in the activity of the corrugator supercilii and procerus muscles at all post‐treatment follow‐up points when compared with baseline. Importantly, no further reductions were observed beyond the 3‐month evaluation, and no differences between treatment groups were detected at any time point. These results reinforce the well‐established mechanism of action of BoNT‐A, namely the inhibition of neuromuscular transmission, leading to decreased muscle contraction [27]. The absence of between‐group differences in EMG activity suggests that the neurophysiological effects of RTU‐aboBoNT‐A and OnaBoNT‐A are equivalent when administered at clinically equivalent recommended doses, in the same clinical settings, as performed in the present study. Unfortunately, there are no previous studies comparing the effects of both formulations using EMG, which limits comparisons of our findings. Interestingly, the neuromuscular effect of RTU‐aboBoNT‐A appears to be more prolonged in women than in men. This observation is supported by a previous study conducted by our group, in which the same dosage regimen applied to glabellar moderate and severe lines resulted in a shorter duration effect—approximately 3 months—in male participants [18]. Although these findings do not allow for direct comparisons or definite conclusions, they suggest the possibility of sex‐related differences in treatment response. One plausible explanation may be the greater baseline muscle strength and contractile capacity typically observed in men, which could lead to a faster recovery of neuromuscular activity.

From a clinical perspective, both formulations resulted in significant and sustained reductions in glabellar line severity during maximum contraction, as assessed by both patients and evaluators, up to 4 months post‐treatment, which aligns with previous studies [14, 17, 28]. At the 5‐month follow‐up, a significant improvement relative to baseline persisted only in patient‐reported assessments, whereas evaluator ratings no longer demonstrated statistical significance. This discrepancy may reflect heightened patient sensitivity to subtle residual effects—considering that muscle EMG activity was still diminished at the 5‐month follow‐up—or subjective expectations, rather than a true difference in objective wrinkle severity. Notably, no differences between treatment groups were observed at any assessment point, either at rest or during contraction, reinforcing the comparable clinical efficacy of RTU‐aboBoNT‐A and OnaBoNT‐A. Importantly, no significant improvements were observed in glabellar line severity at rest, reinforcing the established concept that static lines are less responsive to neuromodulation alone and may require combined approaches targeting different tissue layers to achieve clinically meaningful improvement [29].

Patient satisfaction improved significantly over time in both groups, highlighting the positive perceived impact of glabellar treatment regardless of formulation. A transient between‐group difference was observed at the 2‐month follow‐up, with higher satisfaction scores in the OnaBoNT‐A group. This isolated finding should be interpreted with caution, as satisfaction scores were comparable at all other time points and no parallel differences were observed in objective clinical outcomes. Overall improvements in the appearance of glabellar lines suggest that both formulations effectively addressed a primary goal of aesthetic treatment—enhancing patient satisfaction and perceived well‐being [30] as reported in other clinical trials [15, 17, 18]. Notably, no changes in perceived age were detected within or between groups throughout the study, suggesting that the observed benefits were primarily related to localized aesthetic improvement rather than global facial rejuvenation.

Injection‐related pain emerged as a relevant differentiating factor between formulations. Participants receiving RTU‐aboBoNT‐A reported significantly higher pain scores compared with those treated with OnaBoNT‐A. This finding is consistent with previous reports suggesting that formulation‐specific characteristics, such as differences in pH, osmolarity, and excipient composition, may influence local tolerability [13, 18, 19]. In addition, these findings align with a randomized triple‐blinded clinical trial that also demonstrated higher pain intensity when RTU‐aboBoNT‐A was applied compared with its counterpart abobotulinumtoxinA [18]. Although injection techniques and procedural variables were standardized and performed by an experienced injector in both studies, individual pain perception and tissue sensitivity may also have contributed [31]. These results emphasize that, despite similar efficacy, patient comfort may vary between formulations and should be considered when tailoring treatment choices.

Regarding safety, both treatments were well tolerated, with no serious adverse events reported. Mild and transient local reactions were observed exclusively in the RTU‐aboBoNT‐A group, including itching, cutaneous rash, and under‐eye bags, all of which resolved spontaneously without intervention. These findings are consistent with the known safety profile of BoNT‐A and do not raise concerns regarding the overall clinical use of RTU‐aboBoNT‐A [17, 18].

From a practical standpoint, RTU‐aboBoNT‐A offers logistical advantages, including elimination of the reconstitution step, reduced risk of preparation errors, and potential time savings in clinical practice. Furthermore, one study reported a mean reduction of approximately 1 min in preparation time when comparing RTUaboBoNT‐A with powder BoNT‐A is −1 min [17]. This finding raises an important clinical question: is a 1‐min difference truly clinically relevant? While such a time reduction may appear negligible in isolated procedures, the mentioned benefits may be particularly relevant in high‐volume clinical settings. The higher injection‐related pain and the absence of superior efficacy compared with OnaBoNT‐A suggest that RTUaboBoNT‐A adoption should be based on a balanced appraisal of workflow efficiency, patient comfort, and cost‐related factors, rather than logistical convenience alone (not reconstitution requirement).

Several limitations should be acknowledged. Although the study included only women, which improves internal consistency, the findings cannot be extrapolated to male populations, in whom dosing strategies and treatment durability may differ. Cost‐effectiveness was not evaluated, despite its relevance for real‐world implementation. Finally, while follow‐up extended to 5 months, longer‐term objective assessments could provide further insight into durability differences between formulations. Our study could not performed a longer follow‐up due to dropouts, but previous studies reported RTUaboBoNT‐A long‐term effects (up to 6 months) on glabellar severity wrinkles and patients' satisfaction with the treatment [14, 16]. Also, we should consider that there is not a validated doses equivalence between BoNT‐A trademarks; however, for this study, we used a reported equivalence [32, 33] of 2.5sU for 1 U and since we did not find significant differences in the neuromuscular and subjective assessments, it might be possible that the doses equivalence did not significantly affect our results. Despite these limitations, the study is strengthened by its randomized, triple blinded design and the integration of objective electromyographic data with clinical and patient‐reported outcomes, which is not common in aesthetic studies. Also, this study is the first randomized clinical trial comparing and reporting the effectiveness of the two assessed formulations. Collectively, the findings contribute robust evidence supporting the clinical equivalence of RTU‐aboBoNT‐A and OnaBoNT‐A for the treatment of glabellar lines. Future studies should explore longer follow‐up periods, include economic evaluations and objective assessments, and assess broader patient populations (compare men and women samples) to further inform individualized treatment selection in aesthetic practice.

## 5 Conclusion

Viewed through both objective and patient‐centered outcomes, RTU‐aboBoNT‐A and OnaBoNT‐A demonstrated comparable neuromuscular and clinical efficacy in the treatment of dynamic glabellar lines. Considering RTU‐aboBoNT‐A's higher injection‐related pain, formulation selection should be guided by a balanced evaluation of efficacy, patient comfort and practical considerations.

## Author Contributions

Conceptualization J.R.G. and G.D.T.C.; data curation J.R.G. and L.F.V.; formal analysis G.D.T.C., and A.S.‐A.; investigation J.R.G., A.C.L.N.S., A.C.C. and G.D.T.C.; methodology, J.R.G., L.G.V., A.S.‐A., M.B.C.‐S. and G.D.T.C.; project administration, G.D.T.C. and M.B.C.‐S.; writing – original draft, G.D.T.C., A.C.C. and A.S.‐A.; writing – review and editing, A.C.C., A.S.‐A., M.B.C.‐S. and G.D.T.C. All authors have read and agreed with the final version of the manuscript.

## Funding

This work was supported by FCT—Fundação para a Ciência e Tecnologia, I.P. by project reference UID/4585/2025 (https://doi.org/10.54499/UID/04585/2025).

## Ethics Statement

This randomized, triple‐blind clinical trial was conducted in accordance with the Declaration of Helsinki and received approval from the Research Ethics Committee of Uningá University (CAAE: 83084224.4.0000.5220). All participants were asked to provide a signed written consent to take participate in the study.

## Conflicts of Interest

The authors Ana Claudia Carbone works as a speaker for Galderma—Brazil. All the other authors declare that they have no competing interests.

## Data Availability

All data are available upon reasonable request to the corresponding author.
